# Fog collection on a superhydrophobic/hydrophilic composite spine surface†

**DOI:** 10.1039/d0ra00239a

**Published:** 2020-03-04

**Authors:** Qier An, Jinshu Wang, Feng Zhao, Lei Wang

**Affiliations:** Key Laboratory of Advanced Functional Materials of Education Ministry of China, School of Materials Science and Engineering, Beijing University of Technology 100 Pingleyuan, Chaoyang District Beijing 100124 P. R. China wangjsh@bjut.edu.cn; Department of Solar Energy Engineering, Hainan Vocational University of Science and Technology Haikou 571126 China; Beijing Key Lab of Cryo-biomedical Engineering and Key Lab of Cryogenics, Technical Institute of Physics and Chemistry, Chinese Academy of Sciences 100190 Beijing P. R. China leiwang@mail.ipc.ac.cn

## Abstract

Inspired by numerous plants and animals living in arid conditions, a composite surface with the fog collection capacity has been fabricated in this study. The surface is composed of polydimethylsiloxane-based spine-arrays and a ZnO micron structure. Two wetting properties are integrated on the surface of the spine structure; the tip of spine is processed as hydrophilic and other parts such as the root region of spine and the base are processed as superhydrophobic. When the surface is in the saturated fog flow with a specific tilt angle, the fog deposits on spines and forms condensed droplets; then, the droplets fall off the surface due to gravity. Further, a new cycle of fog collection begins. In this study, we find that the percentage of the hydrophilic tip in the overall spine structure length, the distance between two spines and the tilt angle of surface are the key factors for improving the efficiency of fog collection. Such a composite surface might be an ideal platform for fog collection from air.

## Abstract 2

The fog collection phenomenon on the superhydrophobic/hydrophilic composite spine structure.

## Graphical abstract

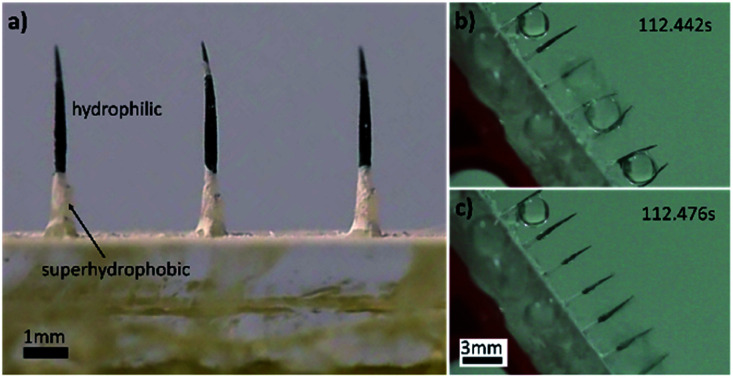

## 1. Introduction

To survive in an arid environment, numerous plants and animals evolved special surface structures, facilitating the collection of water from the fog.^1–5^ A cactus spine can collect water from air due to the gradient of Laplace pressure on its hydrophilic spine.^3,4^ Spider silks can also harvest water from humid air using periodic spindle knots.^6^ Desert beetles utilize their back, which is equipped with hydrophobic and hydrophilic patterns to collect water from fog.^7^ Taking inspiration from these unique creatures, many fog collectors based on anisotropy structure surfaces and structured fibers have been developed.^8–11^

In this study, we prepared a composite surface with clusters of spines and a ZnO micron structure, which perform fog collection. The idea of the spine structure comes from cactus, which can collect water from air with the gradient of Laplace pressure on its spine.^3^ In our system, the spine structure is a simple 3D anisotropy structure made of polydimethylsiloxane (PDMS) through a soft lithographic method. The ZnO micron structure is used to modify the spine structure for improving the surface roughness.^12–16^ ZnO is a usual metallic oxide with the advantages of being low-cost, pollution-free, and higher stability, and also grows easily on the PDMS surface and forms a variety of micron structures *via* a hydrothermal treatment.^17,18^ The temperature of the hydrothermal treatment was lower than 100 °C, which could not damage the PDMS surface.

Moreover, previous studies have demonstrated that the composite structures of hydrophobic and hydrophilic play another significant role in designing a fog collection topography, such as in desert beetles. Inspired by the fog collection performance on the back of a desert beetle,^19^ the tip of spine was made hydrophilic, while the base part and the surface of substrate were made superhydrophobic. In the previous studies on spine structures, fog droplets move to the base of spine due to the gradient of Laplace pressure, and get collected by the hydrophilic substrate.^20^ In our study, we have modified the spine with two wetting properties. The fog droplets easily fall off from the spine. Then, the surface of the substrate remains dry, which induce the circle of fog collection faster. In this way, a highly efficient fog collection surface with a hydrophilic tip and a hydrophobic base is achieved.

## 2. Experimental section

### 2.1 Fabrication of the spine structure surface

**Fig. 1:**
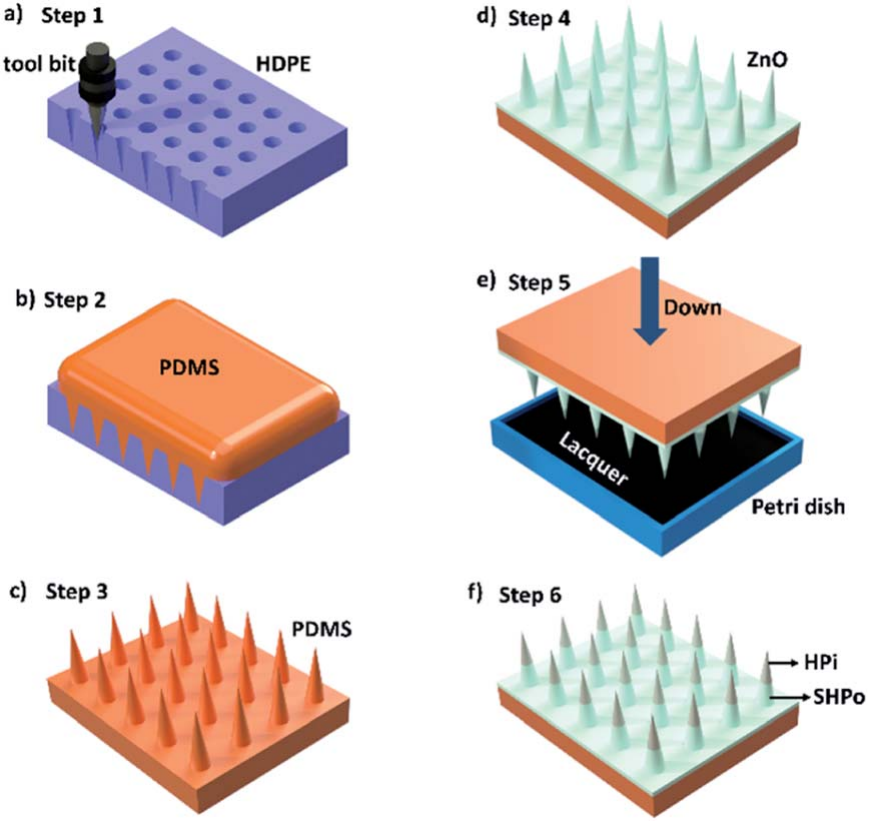
Fabrication of the composite spine structure surface (CS-surface). (a) The tool bit is pressed on an HDPE plate to get an HDPE spine-like opposite structure. (b) The PDMS and the curing agent with a mass ratio of 10 : 1 are mixed and poured onto the HDPE opposite structure. (c) The spine structure surface (S-surface) was achieved. (d) The superhydrophobic S-surface is achieved *via* a hydrothermal treatment. (e) The superhydrophobic S-surface is immobilized on a lifting platform as the tip of spines pointing down, and the hydrophilic lacquer was dumped into a Petri dish, moving the lifting platform until the tip of spines are immersed in the hydrophilic lacquer. (f) The composite spine structure surface (CS-surface), the tip of spine (black) was processed as hydrophilic, while the base part and surface (white) was processed as superhydrophobic.

A spine-like tool bit was installed on a 3D machine tool. Then, the tool bit was pressed on a high-density polyethylene (HDPE) plate to get an HDPE spine-like opposite structure, as shown in Fig. 1a. PDMS (C_2_H_6_OSi)_*n*_ (Dow Chemical Company, 99.99%) and the curing agent (Dow Chemical Company, 99.99%) with a mass ratio of 10 : 1 were mixed and poured on the HDPE spine-like opposite structure. The opposite structure was placed in a vacuum drying oven for 20 min to remove the bubbles. Further, the sample was heated at the temperature of 80 °C for 1 h. The spine structure surface was achieved after peeling off from the template (as shown in Fig. 1b and c).

### 2.2 Fabrication of the liquid metal

45 g of gallium (Sigma-Aldrich, 99.99%) and 5 g of indium (Sigma-Aldrich, 99.99%) were mixed together in a beaker and stirred for 1 h at 150 °C. Then, a uniform mixing metal liquid alloy was obtained. Furthermore, after cooling down to room temperature, the liquid metal (GaIn_10_ alloy) was synthetized.

### 2.3 Fabrication of the composite spine structure surface (CS-surface)

A liquid metal (GaIn_10_ alloy) was painted on the spine structure surface as the crystal nucleus. In traditional crystal growth methods, crystal seed solution need to be heated at high temperature to produce crystal seed on the surface. The liquid metal oxidation layer formed easily on the spine structure surface as crystal seed at room temperature, which keeps the base materials from high temperature in the fabrication of nano-materials.^21,22^ Also, the liquid metal is a kind of alloy with outstanding fluidity under room temperature.^21,22^ So, it is conveniently painted on the uneven spine structure surface. Next, 0.22 g of urea (Sinopharm Chemical Reagent Co., Ltd, 99.99%) and 0.74 g of Zn(NO_3_)_2_·6H_2_O (Sinopharm Chemical Reagent Co., Ltd, 99.99%) were dissolved in 100 mL of deionized water and stirred for 10 min until the solution turned transparent. The solution and the S-surface were transferred into a Teflon-lined stainless-steel autoclave and heated at 90 °C for 12 h. After the hydrothermal treatment, the spine structure substrate with ZnO nanometer-rods was obtained. The ZnO surface was moved into in a vacuum drier with one droplet of silicon tetrafluoride (Sigma Aldrich, 99.99%), and was vacuumed and heated at 80 °C for 12 h to obtain a superhydrophobic plane (shown in Fig. 1d).

#### Synthesis of a hydrophilic lacquer

10 mL of a water base lacquer (Tamiya, 99%), 5 mL of 2-acetoxy-1-methoxypropane (Sinopharm Chemical Reagent Co., Ltd, 99.99%), 5 mL of deionized water and 0.1 g of SDS (sodium dodecyl sulfate, Sinopharm Chemical Reagent Co., Ltd, 99.99%) were mixed and stirred for 20 min for obtaining the hydrophilic lacquer. The superhydrophobic S-surface was immobilized on a lifting platform as the tip of spines pointing down and the hydrophilic lacquer was dumped into a Petri dish (shown in Fig. 1e). Then, the lifting platform was moved until the tip of spines immersed in the hydrophilic lacquer, and dried at room temperature for 0.5 h. Thus, the composite spine structure surface (CS-surface) was obtained, as shown in Fig. 1f.

### 2.4 Characterization

The topography details were obtained *via* scanning electron microscopy (SEM, Hitachi SU8020) under the voltage of 10 kV. Camera photos were taken by a digital camera (Canon EOS 70D).

### 2.5 Measurement of wettability

A PDMS plane was cut to be used as substrate; through the hydrothermal treatment (see Experimental section 2.3), the PDMS plane covered with ZnO nanorods was obtained. The plane was moved into a vacuum drier with one droplet of silicon tetrafluoride (Sigma Aldrich, 99.99%). After being vacuumed and heated at 80 °C for 12 h, the superhydrophobic plane was obtained. 5 mL of the hydrophilic lacquer (see Experimental section 2.3) was moved into a spray gun, and then the lacquer was sprayed (pressure 0.1 Mpa) on the plane to obtain the hydrophilic plane.

### 2.6 Fog collection

The CS-surface was placed in a transparent plexiglass box (size 40 × 30 × 30 cm) with saturated fog flow (humidity 100% by an ultrasonic humidifier) at a temperature of 24 °C. The fog collection phenomenon was recorded using a digital camera (Canon EOS 70D).

### 2.7 Quantitative experiment

The CS-surfaces with different parameters were cut as 30 × 2 mm. Then they were placed in the same plexiglass box with the same experimental conditions as Section 2.6. The volume of collected droplets was measured using two methods: (1) observed and calculated the size and number of droplets by digital camera (Canon EOS 80D). (2) Measured the volume of droplets with a measuring cylinder.

## 3. Results and discussion

**Fig. 2:**
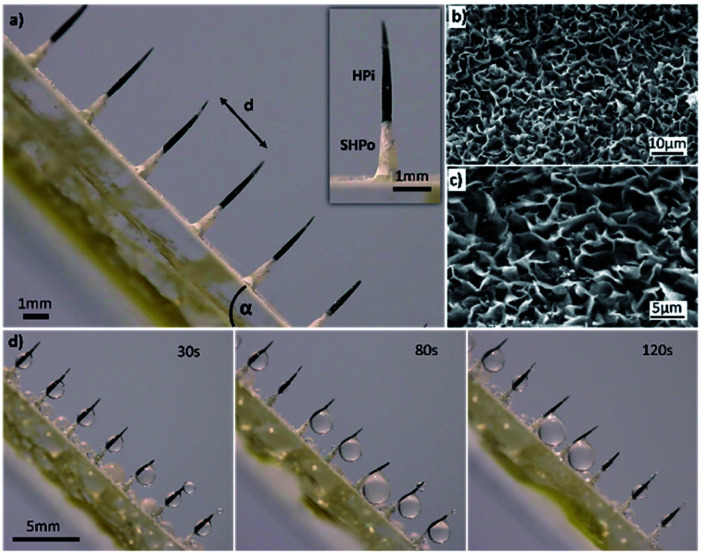
(a) The optical images of the composite spine structure surface (CS-surface). Spine-like structure array on the surface with controllable distances (*d*) ranging from 1 to 3 mm, height of about 4.2 mm and diameter of about 300 μm. The tip of spine (black) is processed as hydrophilic (HPi), and the base part and surface (white) is processed as superhydrophobic (SHPo). (b and c) The SEM images of the ZnO microchip structure. The microchip with a thickness of 300 nm and a height of 1.5 um is observed. (d) The fog collection ability of the CS-surface. The fog coalesces to form droplets on the hydrophilic tip of spines, and the growing droplets move towards the base of spines and then fall off the surface.

The optical images of composite spine structure surfaces (CS-surface) are shown in Fig. 2a. A spine-like structure array was placed on the surface with controllable distances ranging from 1 mm to 3 mm, height of 4.2 mm and diameter of 300 μm. The SEM images of ZnO micron structures are shown in Fig. 2b and c. The tip of spine (black) was processed as hydrophilic, while the base part and surface (white) was processed as superhydrophobic (Fig. 2a).

The measurement of the wettability of the CS-surface was reflected by the planes in Experimental section 2.5. As for the superhydrophobic plane, the contact angle of the water was 151° ± 2.2°. For the hydrophilic plane, the contact angle of the water was 32° ± 1.8° (shown in Fig. S1a and b,† respectively).

The fog collection ability of the CS-surface was investigated using a saturate fog flow (see Experimental section 2.6). The CS-surface with a tilt angle is shown in Fig. 2d, the percentage of the hydrophilic tip (PHT) in the overall spine structure length is 70%, and the distance (*d*) between two spines is 3 mm. At time of 30 s, the fog was collected and formed droplets on the hydrophilic tip of spines. When time was 120 s, the growing droplets moved towards the base of spines and then fell off the surface due to gravity. After the droplets had moved away from the surface, a new cycle of water deposition and collection began.

**Fig. 3:**
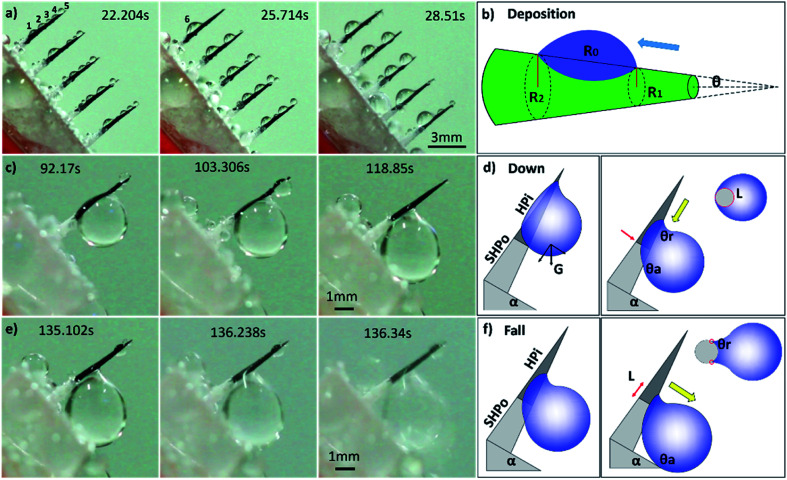
(a and b) The “deposition” process. The fog deposits on the hydrophilic part and forms tiny water droplets 1–5. The gradient of the Laplace pressure between the two sides causes droplets to move towards the base of the spine, and they coalesce to form a larger droplet 6. (c and d) The “down” process. A big droplet moves to the base of spine and touches the superhydrophobic surface because of gravity. (e and f) The “fall” process. The droplet subsequently falls off the CS-surface because of gravity.

For more details on the fog deposition and collection phenomenon, further experiment has been shown in Fig. 3. The CS-surface was placed at a tilt angle (55°), with the PHT of 70%. First, the fog deposited on the hydrophilic part and formed tiny water droplets. As the deposition proceeded, growing droplets 1–5 are shown in Fig. 3a. They moved towards the base of the spine and formed a larger droplet 6, and this is called “deposition” process. During this process, the gradient of the Laplace pressure between two sides induced the droplets on the spines to continuously move towards the base of the spines. This type of conical shape generated a Laplace pressure difference between the two opposite sides of the droplet:^23–25^1
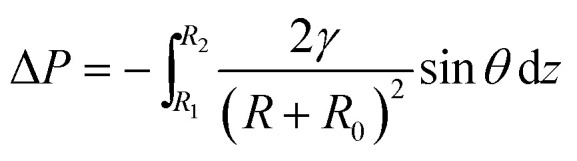
where *R* is the local radius of the spine (*R*_1_ and *R*_2_ are the local radii of the spine at the two opposite sides of the droplet, respectively), *R*_0_ is the drop radius, *γ* is the surface tension of water, *θ* is the half-apex angle of the conical spine, and d*z* is the incremental radius of the spine (as shown in Fig. 3b). The Laplace pressure on the forepart of spine (small radius *R*_1_) was larger than that on the base (large radius *R*_2_). This pressure differential (Δ*P*) within the droplet initiated a driving force that made the tiny droplets to move from the tip to the base side along the spine and made up a big droplet.

First, we studied the fog collection phenomenon on a single spine structure (Fig. 3c–f). The research object was placed at a tilt angle of 55°, with PHT of 70%. After the “deposition” process, with the growing of droplet, big droplet moved to the base of spine and touched the superhydrophobic surface (shown in Fig. 3c). This movement was called the “down” process, and gravity now led the movement of droplet. During the “down” process, the movement of droplet would be hindered while crossing the hydrophilic-superhydrophobic border; however, when gravity and Δ*P* are larger than resistance, the “down” process continued without any hindrance:^26,27^

2 *F*_down_ = *Lγ*(cos *θ*_r_ − cos *θ*_a_)

3 *ρVg* cos *α* + Δ*P* > *Lγ*(cos *θ*_r_ − cos *θ*_a_)

where *F*_down_ is the resistance on the hydrophilic–superhydrophobic border, *L* is the length of the three-phase contact-line (TCL), *γ* is the surface tension of water, *θ*_r_ is the receding angle of droplets and *θ*_a_ is the advancing angle of droplets (shown in Fig. 3d). *ρ* and *V* are the density and volume of droplet, respectively, *α* is the tilt angle, and Δ*P* is the Laplace pressure difference.

After the “down” process, droplet subsequently fell off the CS-surface (“fall” process) when growing beyond a threshold volume where the droplet weight exceeded the adhesive force on the hydrophilic tip (shown in Fig. 3e and f):^26,27^

4 *F*_fall_ = 2*Lγ*(cos *θ*_r_ − cos *θ*_a_)

5 *ρVg* sin *α* > 2*Lγ*(cos *θ*_r_ − cos *θ*_a_)

where *F*_fall_ is the adhesive force of droplets on the hydrophilic tip. The length of TCL (*L*) and volume of droplet (*V*) depended on the PHT of spine. From formulae (2)–(5), the tilt angle *α* and the PHT were the main factors of “down” and “fall” processes. The success of the fog collection depended on the smooth running of the “down” and “fall” processes.

**Fig. 4:**
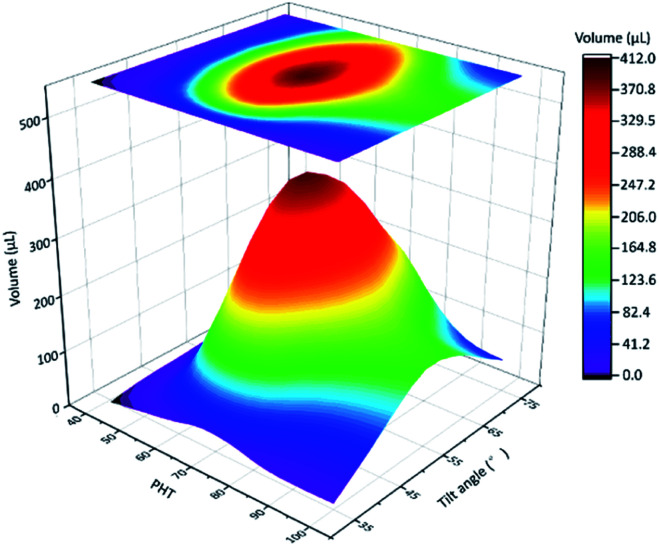
The results of quantitative experiment, the fog collection performance of a single spine with different PHTs and tilt angles. When tilt angle is 55°, PHT is 70%, and the fog collection performance got better.

In the next quantitative experiment, the fog collection performance of a single spine with different PHTs and tilt angles were tested. The samples were placed in a transparent plexiglass box with saturated fog flow, the efficiency of fog collection was measured by the volume of collected droplets in 15 min. The results are shown in Fig. 4, when the tilt angle is closer to 55°, PHT is closer to 70%, and the fog collection performance got better. From formulae (2)–(5), to ensure the function of the “down” and “fall” processes, the tilt angle was 55°, which was the same as the quantitative experiment, and the departing volume of droplet was 10.75 ± 1.56 μL. As for the research object with tilt angle was 55°, the PHT = 70% in Fig. 3c and e, the volume of droplet was 12.77 ± 1.32 μL when it fell off the CS-surface. We got the optimal fog collection performance on this sample, and this result is consistent with that of the quantitative experiment shown in Fig. 4.

In the result of quantitative experiment shown in Fig. 4, when PHT was lower than 70%, there was a significant decrease in efficiency. Lower PHT means smaller droplets, and gravity had a hard time overcoming the surface tension. The “down” and “fall” processes were hard to move on, such as for sample with PHT = 40%, as shown in Fig. S2a.† Moreover, when PHT was higher than 70%, the efficiency was also decreased. As for sample with PHT = 90% shown in Fig. S2b,† the volume of the maximum droplet was 13.45 ± 1.21 μL, the length of TCL was much longer because of higher PHT, and the adhesive force also became larger. From the formula (5), the departing volume of a droplet was 25.08 ± 3.04 μL, resulting in a difficultly of the “fall” process. As for a single spine structure, when the tilt angle was 55°, PHT was 70%, the efficiency of fog collection got better.

Next, we inspected the length of single spine structures, and the fog collection performance of single spine structures with different lengths was tested. The tilt angle was 55° and the PHT was 70%. The test time of fog collection was 15 min. The results are shown in Fig. S3a.† When the length of spine increased form 1.2 mm to 4.2 mm, the efficiency of fog collection increased. When the length of spine was longer than 4.2 mm, the efficiency of fog collection was almost constant. The volume of the maximum droplet during the experiment is shown in Fig. S3b.† The length of spine increased from 1.2 mm to 4.2 mm, the volume of the maximum droplet increased. When the length of spine reached 4.2 mm, the volume of the maximum droplet became 12.77 ± 1.32 μL. In this range, the droplet easy fell off the spine. When the length of spine became longer, the volume of the maximum droplet was almost constant. Due to the results of Fig. S3,† the appropriate length of spine was 4.2 mm, and a longer spine was unnecessary.

**Fig. 5:**
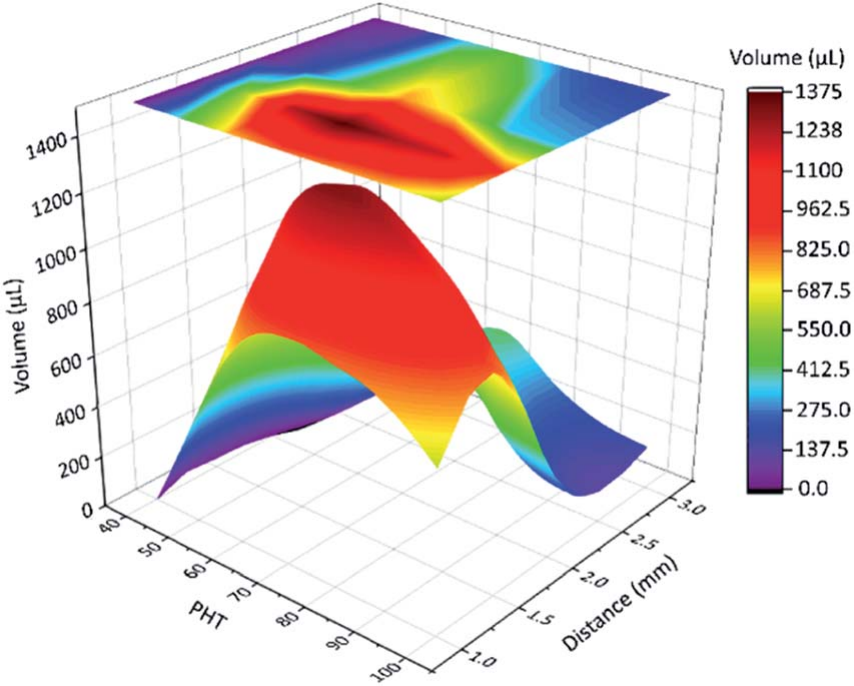
The results of the quantitative experiment, the fog collection performance of a spine structure array with different distances between two spines and PHT. The tilt angle is 55°, the length of spine is 4.2 mm. When PHT is larger than 60%, distance is between 2 mm and 1 mm, the fog collection performance got better, particularly when PHT = 70% and *d* = 1.5 mm.

Next, we focused on the spine structure array. From the above-mentioned results, the tilt angle is 55°, and the length of spines is 4.2 mm. The distance (*d*) between two spines and PHT were the key parameters of fog collection performance. The results of quantitative experiment (see Experimental section 2.7) are shown in Fig. 5 and Table S1.† When PHT was larger than 60%, *d* was between 2 mm and 1 mm, and the fog collection performance got better, particularly when PHT = 70% and *d* = 1.5 mm. As shown in Fig. S2 and S3,† the maximum diameter of droplets in the “fall” process are smaller than 3 mm. When *d* is 3 mm, there were no interactions between droplets on each spine before the “fall” process, and the efficiency of fog collection became mediocre.

**Fig. 6:**
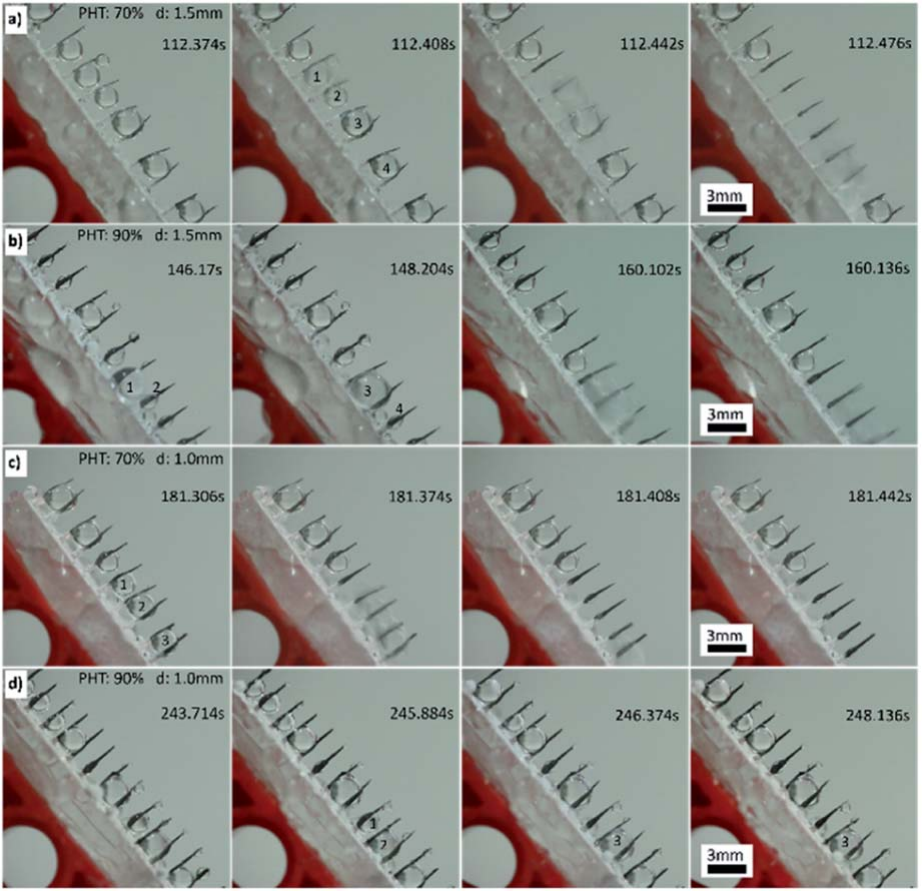
(a) The fog collection phenomenon for sample 70-1.5. Droplets 1–5 coalesce to a bigger droplet and fall off the CS-surface. (b) The fog collection phenomenon for sample 90-1.5. Droplets 1 and 2 coalesce to droplet 3, then droplets 3 coalesces with droplets 4, they fall off the CS-surface. (c) The fog collection phenomenon for sample 70-1.0. Droplets 1–3 coalesce to a big droplet and fall off the CS-surface. (d) The fog collection phenomenon of sample 90-1.0. Droplets 1 and 2 coalesce to droplet 3, droplet 3 is trapped between two spines.

When distance (*d*) was between 2 mm and 1 mm, PHT was larger than 60%, with the proceeding of the “down” and “fall” processes, and the droplets coalesced with their neighbours; thus, the efficiency of fog collection got better, particularly when *d* = 1.5 mm and PHT = 70%. For samples 70-1.5 (PHT = 70%, *d* = 1.5 mm) and 90-1.5 (PHT = 90%, *d* = 1.5 mm), as shown in Fig. 6a (Movie 1†) and Fig. 6b, with the proceeding of “down” and “fall” processes, the droplets coalesced with their neighbours and formed larger droplets and then fell off the CS-surface. For samples 70-1.0 (PHT = 70%, *d* = 1.0 mm) and 90-1.0 (PHT = 90%, *d* = 1.0 mm), as shown in Fig. 6c and d, the coalescence phenomenon for some droplets are not apparent, particularly for 90-1.0, droplets 1 and 2 coalesced to droplets 3, and then got trapped between two spines (Fig. 6d).

With the growth of droplets, they coalesced with their neighbours and formed the larger droplets (shown in Fig. S4a†):

6 *nρVg* sin *α* > 2*Lγ*(cos *θ*_r_ − cos *θ*_a_)

where *n* is the number of droplets that participated in the coalescence process, while other factors are the same as those in formulae (4) and (5). Like samples with PHT > 60%, *d* = 1.5 mm, especially 70-1.5. Most of droplets participated in the coalesce process, the gravity of coalesced big droplets was large enough to overcome the adhesive force, resulting in the higher efficiency of fog collection. When *d* was below 1 mm, the droplets coalesced prematurely, and the gravity of coalesced droplets was not enough to overcome the surface tension; as a result, many coalesced droplets got trapped between two spines (Fig. S4b†) and could not fell off, which induced the lower efficiency of fog collection. As *d* increased to 3 mm, the coalescence process did not occur, *n* = 1, each droplet fell individually, the efficiency of fog collection was lowest. When PHT was too low (lower than 60%), the gravity of droplets was too small to overcome the surface tension even when they coalesced. Fig. S5† shows the fog collection performance of sample 40-1.0 (PHT = 40%, *d* = 1.0 mm), and the coalesced droplets were trapped between more than two spines and were difficult to grow and fall. Thus, the fog collection efficiency was almost zero.

From what had been discussed above, when PHT was larger than 60% and *d* was 1.5 mm, the fog collection performance was better, particularly when PHT = 70%. As shown in further quantitative experiment (Fig. S6†), samples 60-1.5, 70-1.5, 80-1.5, 90-1.5 and 100-1.5 were placed in a transparent plexiglass box with saturated fog flow, the efficiency of fog collection was measured by the volume of collected droplets per 15 min in series of time. After a long test, the efficiency of the fog collection of every sample was stable. Owing to the superhydrophobic substrate, the collected droplets were easy to fall off the surface, and the cycle of fog collection went well.

## 4. Conclusions

In this study, we successfully prepared a composite surface with clusters of spines and a ZnO micron structure for realizing the fog collection capacity. Two wetting properties were integrated on the surface of the spine structure: the tip of spine was processed as hydrophilic, while the base part and substrate were processed as superhydrophobic. The tilt angle of the substrate was 55°, and the length of spine was 4.2 mm. When the percentage of the hydrophilic tip (PHT) in the overall spine structure length was larger than 60%, the distance between two spines was 1.5 mm, and the efficiency of fog collection would be better, particularly when PHT = 70%. In long test, the efficient of fog collection was stable.

## Conflicts of interest

There are no conflicts to declare.

## Supplementary Material

RA-010-D0RA00239A-s001

RA-010-D0RA00239A-s002

## References

[cit1] Kuang M., Wang J., Jiang L. (2016). Chem. Soc. Rev..

[cit2] Liu K., Yao X., Jiang L. (2010). Chem. Soc. Rev..

[cit3] Ju J., Bai H., Zheng Y., Zhao T., Fang R., Jiang L. (2012). Nat. Commun..

[cit4] Ju J., Yao X., Yang S., Wang L., Sun R., He X., Jiang L. (2014). Adv. Funct. Mater..

[cit5] Hao C., Liu Y., Chen X., Li J., Zhang M., Zhao Y., Wang Z. (2016). Small.

[cit6] Zheng Y., Bai H., Huang Z., Tian X., Nie F., Zhao Y., Zhai J., Jiang L. (2010). Nature.

[cit7] Garrod R. P., Harris L. G., Schofield W. C. E., McGettrick J., Ward L. J., Teare D. O. H., Badyal J. P. S. (2007). Langmuir.

[cit8] Azad M. A. K., Barthlott W., Koch K. (2015). Langmuir.

[cit9] Cao M., Xiao J., Yu C., Li K., Jiang L. (2015). Small.

[cit10] Daniel S., Chaudhury M., Chen J. (2001). Science.

[cit11] Ju J., Xiao K., Yao X., Bai H., Jiang L. (2013). Adv. Mater..

[cit12] Yu H., Liu J., Fan X., Yan W., Han L., Han J., Zhang X., Hong T., Liu Z. (2016). Mater. Chem. Phys..

[cit13] Liu Y., Lin Z., Lin W., Moon K., Wong C. (2012). ACS Appl. Mater. Interfaces.

[cit14] Li H., Yu S., Han X. (2015). New J. Chem..

[cit15] Wen M., Wang L., Zhang M., Jiang L., Zheng Y. (2014). ACS Appl. Mater. Interfaces.

[cit16] Lai Y., Lin Z., Huang J., Sun L., Chen Z., Lin C. (2010). New J. Chem..

[cit17] Wang L., Gong Q., Zhan S., Jiang L., Zheng Y. (2016). Adv. Mater..

[cit18] Kumar S., Rao K. (2015). RSC Adv..

[cit19] Wen C., Guo H., Bai H., Xu T., Liu M., Yang J., Zhu Y., Zhao W., Zhang J., Cao M., Zhang L. (2019). ACS Appl. Mater. Interfaces.

[cit20] Guo L., Tang G. H. (2015). Int. J. Heat Mass Transfer.

[cit21] Dickey M., Chiechi R., Larsen R., Weiss E., Weitz D., Whitesides G. (2008). Adv. Funct. Mater..

[cit22] Chiechi R., Weiss E., Dickey M., Whitesides G. (2008). Angew. Chem..

[cit23] Peng Y., He Y., Yang S., Ben S., Cao M., Li K., Liu K., Jiang L. (2015). Adv. Funct. Mater..

[cit24] Lorenceau E., Quere D. (2004). J. Fluid Mech..

[cit25] Wang M., Liu Q., Zhang H., Wang C., Wang L., Xiang B., Fan Y., Guo C., Ruan S. (2017). ACS Appl. Mater. Interfaces.

[cit26] Yang X., Song J., Liu J., Liu X., Jin Z. (2017). Sci. Rep..

[cit27] Malvadkar N., Hancock M., Sekeroglu K., Dressick W., Demirel M. (2010). Nat. Mater..

