# Supplementary material for: Fog collection on a superhydrophobic/hydrophilic composite spine surface
Source: RSC Adv. 2020 Mar 4;10(16):9318–23. doi: 10.1039/d0ra00239a (PMC9050068; doi:10.1039/d0ra00239a)
Supplement: RA-010-D0RA00239A-s001 [file RA-010-D0RA00239A-s001.pdf]

Electronic Supplementary Material (ESI) for RSC Advances.
This journal is © The Royal Society of Chemistry 2020

Fog Collection on Superhydrophobic/ Hydrophilic Composite Spines Surface

Qier An,[a] Jinshu Wang,[a*] feng zhao[b] and Lei Wang[c*]

a Key Laboratory of Advanced Functional Materials of Education Ministry of China, School of Materials Science and Engineering, Beijing University of Technology, 100 Pingleyuan, Chaoyang District, Beijing 100124, Beijing, P. R. China.

b Department of Solar Energy Engineering, Hainan Vocational University of Science and Technology, Haikou 571126, China

c Beijing Key Lab of Cryo-biomedical Engineering and Key Lab of Cryogenics, Technical Institute of Physics and Chemistry, Chinese Academy of Sciences, 100190 Beijing, P. R. China.

E-mail: wangjsh@bjut.edu.cn (J Wang), leiwang@mail.ipc.ac.cn (L Wang)

Electronic Supplementary Material (ESI) for RSC Advances.

Supplementary Figure Legends: (Figure S1-S6)

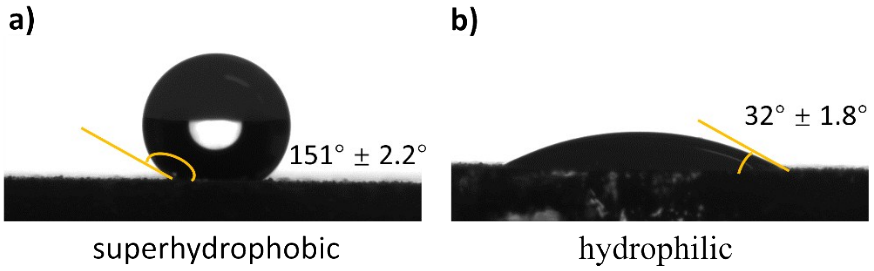

Fig S1. a) Wettability of superhydrophobic plane, the contact angle of the water is 151° ± 2.2°. b) Wettability of hydrophilic plane, the contact angle of the water is 32° ± 1.8°.

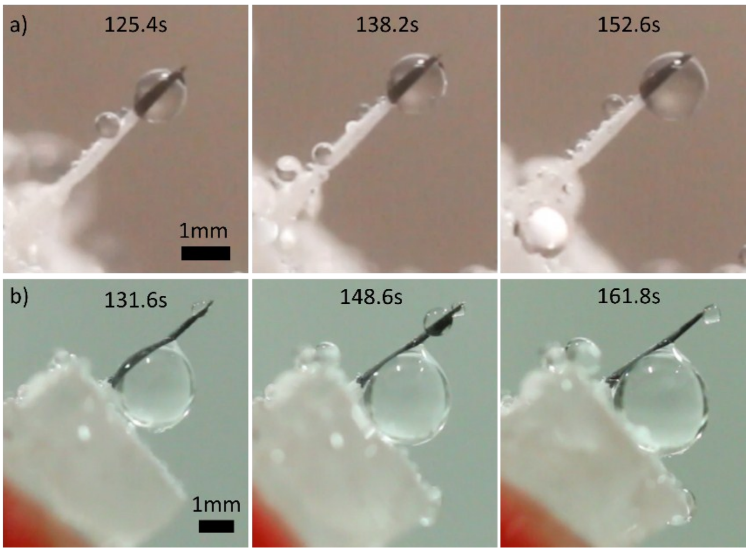

Fig S2. a) The fog collection phenomenon for single spine, tilt angle is 55°, PHT=40%, the droplet is small and difficult to fall. b) The fog collection phenomenon for single spine, tilt angle is 55°, PHT=90%, the adhesive force on hydrophilic tip is lager, droplet cannot fall off the sample.

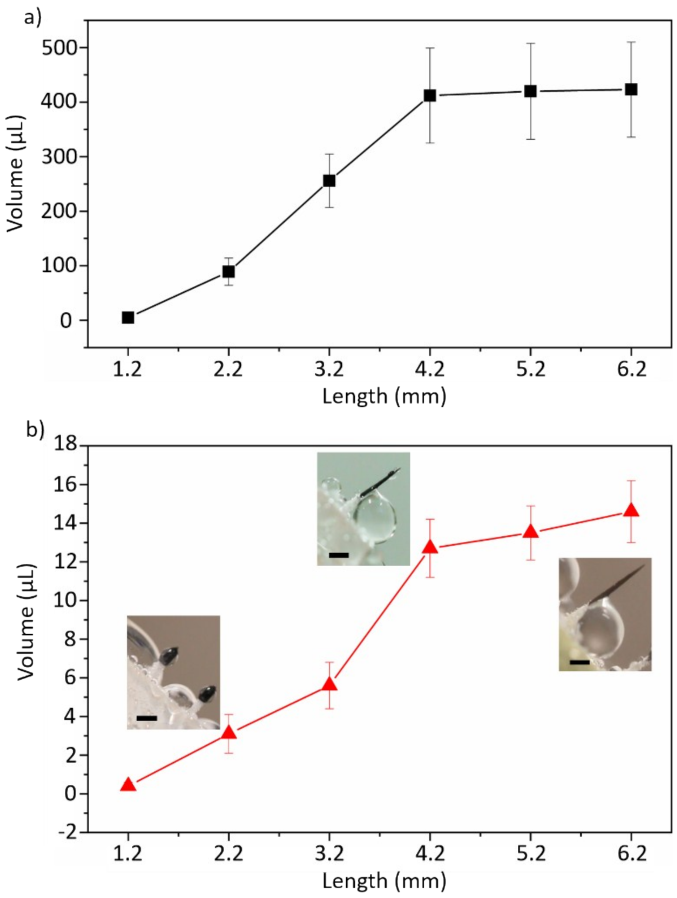

Fig S3. a) The efficiency of fog collection of single spine with different length. When the length of spine increases form 1.2 mm to 4.2 mm, the efficiency of fog collection is increasing. When the length of spine is longer than 4.2mm, the efficiency of fog collection is almost constant. b) The volume of maximum droplet, when the length of spine increasing form 1.2 mm to 4.2 mm, the volume of maximum droplet is increasing. When the length of spine is longer than 4.2mm, the volume of maximum droplet is almost constant.

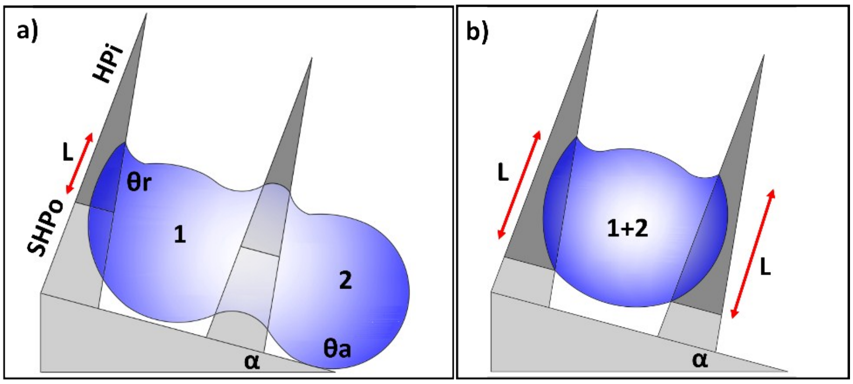

Fig S4. a) The sketch of coalescence phenomenon, with the growing of droplets, they coalesce with their neighbors and form the larger droplets. b) The sketch of coalescence process. When *d* down to 1 mm, coalesced droplets are trapped between two spines.

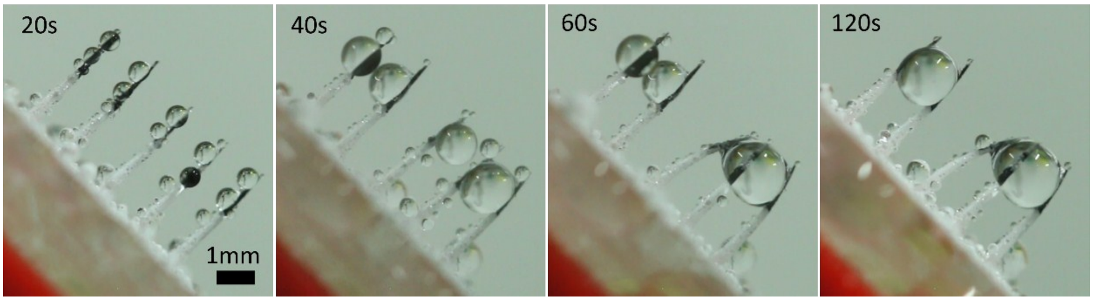

Fig S5. The coalescence phenomenon of sample 40-1.0 (PHT=40%, d=1.0 mm), the coalesced droplets are trapped between more than two spines and difficult to grow and fall.

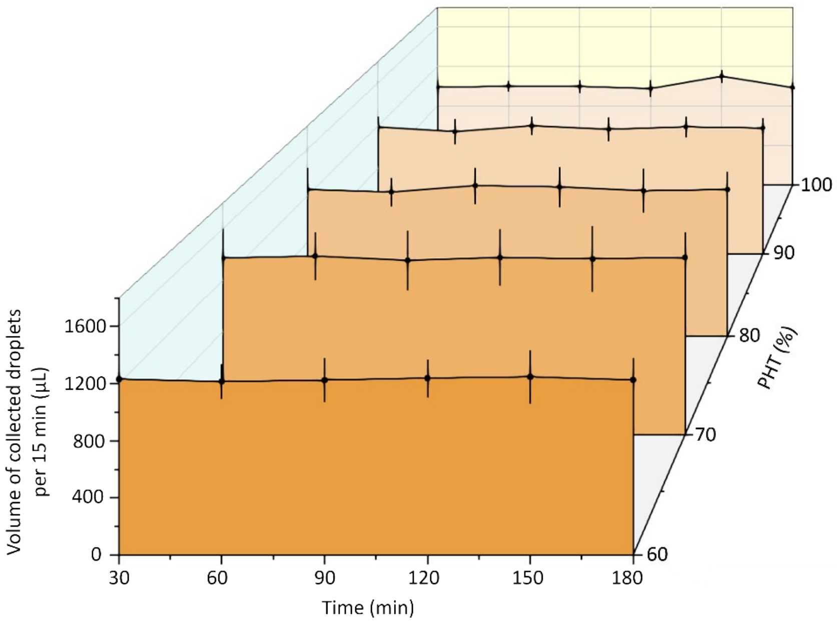

Fig S6. The quantitative experiment of samples 60-1.5, 70-1.5, 80-1.5, 90-1.5 and 100-1.5. After a long test, the efficient of fog collection of every samples are stable.

Table 1

PHT \ d (Volume)	3.0mm	1.5mm	1.0mm
100%	136±47 μL	987±67 μL	682±69 μL
90%	192±67 μL	1189±89 μL	766±84 μL
80%	267±57 μL	1256±235 μL	803±102 μL
70%	455±89 μL	1374±128 μL	834±79 μL
60%	356±61 μL	1232±176 μL	755±98 μL
50%	79±24 μL	576±84 μL	388±115 μL
40%	24±17 μL	0 μL	0 μL

Table S1. The results of quantitative experiment. When the PHT is 60%-100%, the *d* is 1.5 mm, the efficiency of fog collection will be better.
